# A clinical phase I dose-finding design with adaptive shrinking boundaries for drug combination trials

**DOI:** 10.1186/s12874-023-01867-y

**Published:** 2023-03-02

**Authors:** Zhaohang Li, Ze Xu, Aijun Zhang, Guanpeng Qi, Zuojing Li

**Affiliations:** Department of Pharmaceutical Informatics, Academy of Pharmacy, Shenyang Pharmaceutical University, Shenyang, Liaoning China

**Keywords:** Dose escalation, Model-assisted design, Drug combination, Bayesian adaptive dose-finding design, Phase I clinical trial design

## Abstract

**Background:**

Combinations of drugs are becoming increasingly common in oncology treatment. In some cases, patients can benefit from the interaction between two drugs, although there is usually a higher risk of developing toxicity. Due to drug–drug interactions, multidrug combinations often exhibit different toxicity profiles than those of single drugs, leading to a complex trial scenario. Numerous methods have been proposed for the design of phase I drug combination trials. For example, the two-dimensional Bayesian optimal interval design for combination drug (BOINcomb) is simple to implement and has desirable performance. However, in scenarios where the lowest and starting dose is close to being toxic, the BOINcomb design may tend to allocate more patients to overly toxic doses, and select an overly toxic dose combination as the maximum tolerated dose combination.

**Method:**

To improve the performance of BOINcomb in the above extreme scenarios, we widen the range of variation of the boundaries by setting the self-shrinking dose escalation and de-escalation boundaries. We refer to the new design as adaptive shrinking Bayesian optimal interval design for combination drug (asBOINcomb). We conduct a simulation study to evaluate the performance of the proposed design using a real clinical trial example.

**Results:**

Our simulation results show that asBOINcomb is more accurate and stable than BOINcomb, especially in some extreme scenarios. Specifically, in all ten scenarios, the percentage of correct selection is higher than the BOINcomb design within 30 to 60 patients.

**Conclusion:**

The proposed asBOINcomb design is transparent and simple to implement and can reduce the trial sample size while maintaining accuracy compared with the BOINcomb design.

## Introduction

**Fig. 1:**
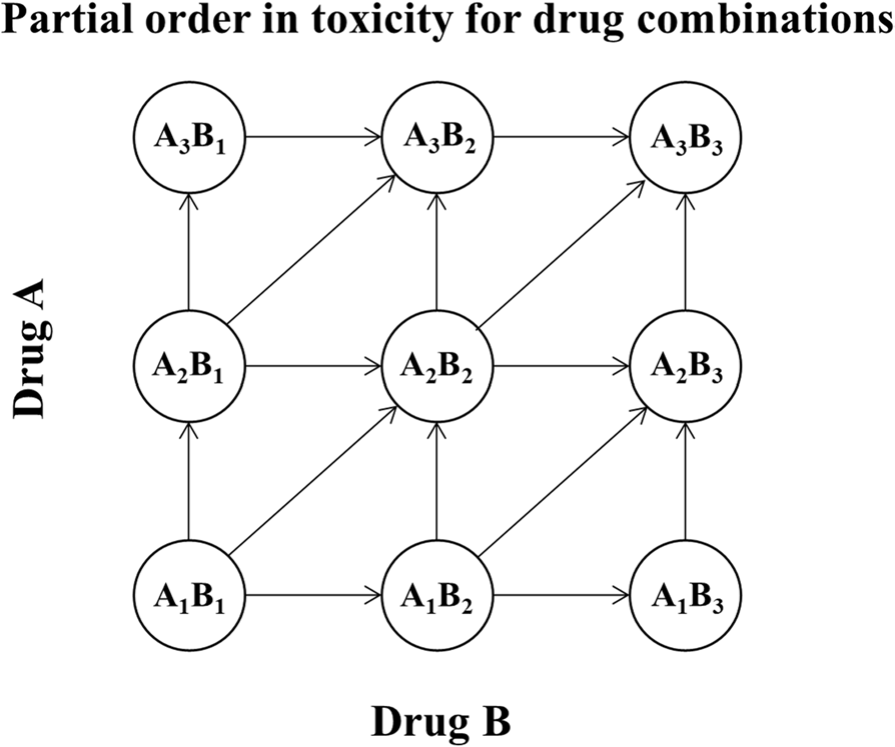
Partial toxicity order as shown. The probability of toxicity is greater for the A1B2 combination than for A1B1. In drug combination trials, the order of toxicity for all dose combinations is not entirely clear (e.g., between A2B1 and A1B2)

**Fig. 2:**
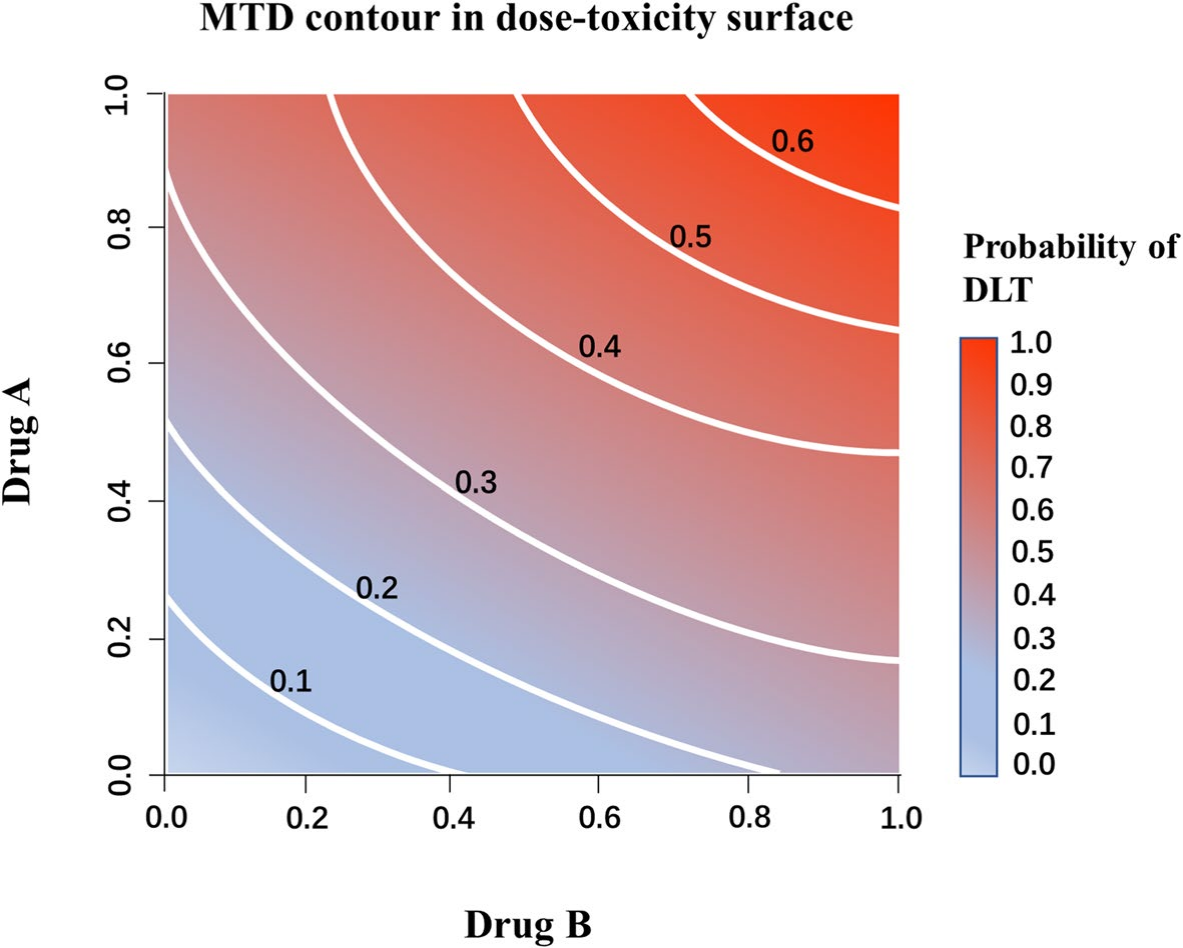
Example of equivalent contour lines based on DLT probability for multiple dose combinations

Drug combination therapy provides an important method for the treatment of difficult diseases such as cancer. The purpose of drug combination therapy is to induce synergistic therapeutic effects, increase the combined dose intensity, and achieve better therapeutic effects without cross-toxicity. The purpose of a drug combination dose escalation trial is to identify the maximum tolerated dose combination (MTDC) based on a prespecified target toxicity rate. In a single-drug dose escalation trial, toxicity is typically assumed to increase with an increasing dose. However, the order of toxicity between dose combinations in two-drug combination trials is only partially known. If the dose of one drug in the combination increases while that of the other drug decreases, it is unknown whether the toxicity increases or decreases (Fig. 1). This partial ordering assumption impacts interim dose allocation decisions. On the other hand, the MTDC may differ from the combination formed by the maximum tolerated dose (MTD) of each drug alone due to unknown potential interaction between the drugs (synergy, antagonism, or no interaction). In fact, multiple dose combinations may have the same target dose-limiting toxicity (DLT) probability, thus yielding an MTDC contour (Fig. 2). Therefore, the traditional single-drug dose-finding design cannot be used directly for combination drug dose-finding trials. The most common method for two-drug combination trials is to fix the dose of one drug and conduct a dose-finding trial on the other drug, i.e., reducing a two-dimensional dose combination-finding problem to a one-dimensional dose-finding one, which largely limits the ability to evaluate potential synergy between drugs. In addition, an increasing number of methods applicable to two-drug combination dose-finding trials have been proposed. These methods can be broadly classified into three categories: algorithm-based design, model-based design, and model-assisted design. Algorithm-based designs rely on several prespecified rules to determine when doses are escalated, de-escalated, or selected as MTDC. Examples include the up-and-down design [1], 2 + 1 + 3 design [2], and 3 + 3 + 3 and its derivative designs [3]. Model-based designs simulate the relationship between dose and toxicity probabilities via parametric functions. During the trial, parameter estimates are continuously updated to better describe this relationship. Thall et al. [4] proposed an adaptive two-stage Bayesian design by considering a six-parameter joint toxicity rate model. Wang and Ivanova developed a method based on a three-parameter model that uses Bayesian inference [5] to estimate parameters. Yin and Yuan [6] used copula-type regression models to relate the toxicity rates of two drugs based on several feasible conditions, which can be viewed as a generalized or two-dimensional version of the continuous reassessment method (CRM) [7]. Wages et al. [8] developed a partially ranked CRM by listing the possible order of the combined toxicity rates. Yin et al. [9] developed a Bayesian adaptive design for dose discovery based on a potential 2 × 2 table. Thomas et al. [10] proposed a hierarchical model for the probability of DLT for dose combinations and applied this model to a Bayesian adaptive trial design. Riviere et al. [11] developed a Bayesian dose discovery design for clinical trials combining cytotoxic drugs with molecularly targeted drugs. Manjrekar et al. [12, 13] proposed using a continuation ratio model to separate the toxicity and efficacy profiles of each drug, and combining them into the optimal dosing region to determine the combination. Li et al. [14] proposed a Bayesian hierarchical model that jointly models the unordered probabilities of toxicity and efficacy in the design of a dose/schedule-finding trial, and applied a Bayesian isotonic transformation to the posterior samples of the toxicity probabilities to impose a partial ordering constraint. Guo et al. [15] developed a dose-schedule-finding algorithm to allocate patients sequentially to a desirable dose–schedule combination and to select an optimal combination at the end of the trial. Model-assisted designs do not prespecify any relationship between dose and toxicity and therefore do not rely on any parametric assumptions when finding the MTDC. However, unlike in algorithm-based designs, the decision process for dose escalation and de-escalation is aided by statistical models. Lin et al. [16] proposed a two-dimensional Bayesian optimal interval design, Zhang et al. [17] proposed a Bayesian optimal interval-based design for exploring multiple MTDCs, and Pan et al. [18] extended the keyboard design to a two-drug combination dose-finding trial. Among them, the two-dimensional Bayesian optimal interval (BOINcomb) design has overall good operating characteristics [16]. The BOINcomb design is also comprehensible and easily implementable as an extension of the one-dimensional Bayesian optimal interval (BOIN) design to the two-drug combination case. However, due to the complexity of two-drug combination trial scenarios, the two-dimensional Bayesian optimal interval design may not perform well in some extreme scenarios and may tend to allocate more patients at overly toxic doses [19]. In practice, extreme scenarios may not be common, but they do arise in real trials [20–22]. For example, in a phase I study of Ganetespib and Ziv-Aflibercept in patients with advanced carcinomas and sarcomas, the lowest and starting dose combination may be adjacent to toxic dose combinations in the dose-combination grid being investigated [22]. To improve the performance, specifically, flexibility and stability, of the BOINcomb design in extreme scenarios, we modify BOINcomb by proposing adaptively-shrinking dose escalation and de-escalation boundaries, the idea of which has been successfully implemented with improvements in design performance demonstrated in one-dimensional phase I dose-finding trials [23, 24] and two-dimensional dose-schedule-finding trials.

The rest of this paper is organized as follows. In Section 2, the BOIN design, the BOINcomb design, and the improved BOINcomb design, denoted as asBOINcomb design, are presented. In Section 3, a simulation study is used to evaluate our proposed design. The results of the simulation study are also analyzed. Section 4 uses a trial example to illustrate our proposed design. Section 5 provides some brief discussions. Finally, a summary is presented in Section 6.

## Methods

### BOIN design

The BOIN (Bayesian optimal interval) design is a Bayesian model-assisted phase I dose-finding design proposed by Liu and Yuan Y (2015). The design is simple and flexible and performs comparably to model-based designs [25]. Dose escalation and de-escalation in this design are determined by comparing the observed DLT rate at the current dose with a fixed pair of dose escalation and de-escalation boundaries. The specific rules are as follows: assume that ${\hat{p}}_j={y}_j/{n}_j$ is the observed DLT probability at the current dose, λ_e_ and λ_d_ denote the predetermined dose escalation and de-escalation boundaries, respectively, and *j* is the current dose level. The BOIN design determines the next dose as follows:

- if ${\hat{p}}_j\le {\lambda}_e$, then escalate the dose level to *j* + 1,
- if ${\hat{p}}_j>{\lambda}_d$, then de-escalate the dose level to *j* – 1,
- otherwise, i.e., *λ*_*e*_ < ${\hat{p}}_j\le {\lambda}_d$, retain the same dose level *j*.

The trial continues until the prespecified sample size is exhausted or the trial is stopped due to excessive toxicity.

The boundary designation algorithm for the BOIN design aims to minimize incorrect decisions on dose assignment. It makes three assumptions:

$${H}_{0j}:{p}_j=\upphi$$

$${H}_{1j}:{p}_j={\upphi}_1$$

$${H}_{2j}:{p}_j={\upphi}_2$$

where *p*_*j*_ denotes the true toxicity probability at dose level *j* (1, 2..., *j*), where ϕ_1_ denotes the highest toxicity probability that is deemed subtherapeutic (i.e., below the MTD) such that dose escalation should be made, and ϕ_2_ denotes the lowest toxicity probability that is deemed overly toxic such that dose deescalation is required.

Under the Bayesian model, the three hypotheses are given equal prior probabilities, denoted as *π*_*kj*_ = Pr(*H*_*kj*_), *k* = 0, 1, 2, and the escalation and de-escalation boundaries are:

1 $${\lambda}_e=\frac{\mathit{\log}\left(\frac{1-{\upphi}_1}{1-\upphi}\right)}{\mathit{\log}\left\{\frac{\upphi \left(1-{\upphi}_1\right)}{\upphi_1\left(1-\upphi \right)}\right\}}$$

2 $${\lambda}_d=\frac{\mathit{\log}\left(\frac{1-\upphi}{1-{\upphi}_2}\right)}{\mathit{\log}\left\{\frac{\upphi_2\left(1-\upphi \right)}{\upphi \left(1-{\upphi}_2\right)}\right\}}$$

The BOIN design is easy to implement and has comparable performance to that of existing one-dimensional phase I dose-finding design [25].

### BOINcomb design

The two-dimensional BOIN design for drug combination trials is based on an extension of the one-dimensional design. The details are as follows. Assume that ${\hat{p}}_{jk}$ denotes the probability of toxicity of two drugs at dose combination (*j*, *k*), 1 ≤ *j* ≤ J,1 ≤ *k* ≤ *K*. Assume that the current dose combination is (*j*, *k*), and let ${\hat{p}}_{jk}$ denote the estimated probability of toxicity based on the cumulative information of the dose combination (*j*, *k*). ${\hat{p}}_{jk}={y}_{jk}/{n}_{jk}$, where *y*_*jk*_ and *n*_*jk*_ denote the number of patients with toxicity and patients treated at that dose combination, respectively. The acceptable dose escalation set is defined as *A*_*E*_ = {(*j* + 1, *k*), (*j*, *k* + 1)}, and the allowable dose de-escalation set is *A*_*D*_ = {(*j* − 1, *k*), (*j*, *k* − 1)}.

The rules of BOINcomb are as follows:

1. Treat the first cohort of subjects with the lowest dose combination (1, 1).
2. Assuming that the current cohort is treated with dose combination (*j*, *k*), then for the next cohort of patients:
  - If ${\hat{p}}_{jk}\le {\lambda}_e$, escalate the dose to the dose combination with the largest value of $\mathit{\Pr}\left\{{p}_{j^{\prime }{k}^{\prime }}\in \left({\lambda}_e,{\lambda}_d\right)|{y}_{j^{\prime }{k}^{\prime }}\right\}$ in *A*_*E*_.
  - If ${\hat{p}}_{jk}\ge {\lambda}_d$, de-escalate the dose to the dose combination with the largest value of $\mathit{\Pr}\left\{{p}_{j^{\prime }{k}^{\prime }}\in \left({\lambda}_e,{\lambda}_d\right)|{y}_{j^{\prime }{k}^{\prime }}\right\}$ in *A*_*D*_.
  - Otherwise, if ${\lambda}_e<{\hat{p}}_{jk}<{\lambda}_d$, the doses remain the same for the combination (*j*, *k*).
3. This process continues until the total sample size is exhausted.

During dose escalation and de-escalation, if there are multiple optimal dose combinations in sets *A*_*E*_ and *A*_*D*_, we randomly choose one with equal probability. If no dose combination exists in sets *A*_*E*_ and *A*_*D*_, we retain the current dose combination. Regarding the encountered boundary case, if *j* = 1 and ${\hat{p}}_{jk}\ge {\lambda}_d$, the next dose combination is (*j*, *k* - 1), and if (*j*, *k*) = (1, 1), the current dose is kept. If *j* = *J* and ${\hat{p}}_{jk}\le {\lambda}_e$, then the next dose combination is (*j*, *k* + 1), and if (*j*, *k*) = (*J*, *K*), then the current dose is maintained. Due to the symmetry between *j* and *k*, the same rules apply to *k*. Each *p*_*jk*_ follows the (noninformative) Jeffreys prior Beta (0.5, 0.5) distribution [26].

### asBOINcomb design

To improve the flexibility and stability of BOINcomb in extreme scenarios, we adopt nonfixed boundaries and introduce sample size n_j_ to dynamically adjust the boundary such that it shrinks with increasing sample size. However, if only this aspect is considered, both dose escalation and de-escalation would be more likely to occur in the later stages of the trial, potentially increasing the number of people treated at high doses. Therefore, we also use parameter *t* to further control the change trend of the boundary and ensure that the initial boundary is the same as in the BOIN design (when n_j_ is 1, ϕ_1_ and ϕ_2_ are the same as in the BOIN design). In the BOIN design framework, we reconstruct ϕ_1_ and ϕ_2_ as

$${\upphi}_1=\upphi -\frac{\Delta _1}{\frac{n_j-1}{t_1}+1}$$

$${\upphi}_2=\upphi +\frac{\Delta _2}{\frac{n_j-1}{t_2}+1}$$

where n_j_ is the cumulative number of patients treated at dose level *j* during the trial and *t*_*1*_,*t*_*2*_ > 0 are two acceleration factors to control the contraction rate of the two boundaries. Parameters ∆_1_ and ∆_2_ are the initial value of the prespecified boundaries, i.e., the initial boundary value when the first patient is enrolled in the trial. Clearly, the two fixed boundaries of the original BOIN design now depend on the dynamic number n_j_, i.e., the number of patients treated at dose level *j*. This method of construction would clearly make ϕ_1_ and ϕ_2_ converge to the MTDC target ϕ as n_j_ increases. This construction is also highly flexible for designing clinical trials. For example, to reduce toxicity, we can penalize the practice of assigning patients to dose levels above the MTDC by using discount parameters t_1_ > t_2_ so that the de-escalation boundary shrinks more rapidly than the escalation boundary. Similar to the BOIN design, the optimal λ_1j_ and λ_2j_ minimize the decision error rate and can be derived as λ_e_(Δ_1_, n_j_) and λ_d_(Δ_2_, n_j_) with the redefined ϕ_1_ and ϕ_2_ plugged into (1) and (2), respectively:

3 $${\lambda}_e=\frac{\mathit{\log}\left\{1+\frac{\Delta _1}{\left(1-\upphi \right)\left(\frac{n_j-1}{t_1}+1\right)}\right\}}{\mathit{\log}\left\{\frac{1+\frac{\Delta _1}{\left(1-\upphi \right)\left(\frac{n_j-1}{t_1}+1\right)}}{1-\frac{\Delta _1}{\upphi \left(\frac{n_j-1}{t_1}+1\right)}}\right\}}$$

4 $${\lambda}_d=\frac{{\mathit{\log}}^{-1}\left\{1-\frac{\Delta _2}{\left(1-\upphi \right)\left(\frac{n_j-1}{t_2}+1\right)}\right\}}{\mathit{\log}\left\{\frac{1+\frac{\Delta _2}{\upphi \left(\frac{n_j-1}{t_2}+1\right)}}{1-\frac{\Delta _2}{\left(1-\upphi \right)\left(\frac{n_j-1}{t_2}+1\right)}}\right\}}$$

where we set a parameter set controlling the boundary shrinkage with thousands of parameter combinations to ensure that the optimal design performance can be achieved in various clinical trial scenarios. The rest of the design rules are the same as in BOINcomb. In contrast to the BOINcomb design, since a dynamic boundary value is adopted in the asBOINcomb design, the dose decision is related not only to the number of patients treated at the current dose but also the total number of patients enrolled in the trial. Moreover, like the BOINcomb design, the asBOINcomb design also has the advantages of transparency and convenience, and the dose decision table can still be generated in advance according to the trial settings.

The parameter *t*_1_*and t*_2_, which control the boundaries, play an important role in a clinical trial. Its value can be determined through simulation and will influence the rate of dose escalation in the trial. When conducting a clinical trial, it is important to carefully select the value of *t*_1_*and t*_2_ in order to ensure the safety and performance. Therefore, when conducting simulation studies to assess the risks of a trial, biostatisticians must collaborate more closely with clinical investigators to assess the risks of trials. In general, when setting the *t* parameters, to further enhance safety of the design, especially in oncology trials, we recommend to choose *t*_*1*_ > *t*_*2*_, the trial will be more conservative in terms of dose escalation, and conversely, a fast rate of dose escalation may increase the toxicity of the trial.

To clarify our design, Table 1 provides examples of the values of (λ_e_ (n_j_), λ_d_(n_j_)) for target ϕ = 0.3.

**Table 1:** Dose escalation and de-escalation boundaries for the asBOINcomb design, with φ = 0.3, t_1_ = t_2_ = 100, Δ_1=_0.3φ, and Δ_2=_1.7φ

n_**j**_	3	6	9	12	15	18	21	24	27	30
**λ**_**e**_	0.179	0.186	0.190	0.194	0.197	0.200	0.203	0.206	0.208	0.211
**λ**_**d**_	0.402	0.397	0.394	0.392	0.389	0.387	0.385	0.383	0.381	0.379

## Simulation studies

### Determination of the boundary range

Due to the dynamic properties of the asBOINcomb boundaries, there may be a better choice than the original BOINcomb recommended boundaries. We conducted a simulation study for the specified values of the default boundaries. In addition to the default values recommended by the original BOINcomb design (∆_1_ = 0.6ϕ, ∆_2_ = 1.4ϕ), we considered eight different sets of values (Table 2).

**Table 2:** Eight different boundary ranges (∆_1_ is the initial value of the increasing boundary and ∆_2_ is the initial value of the decreasing boundary)

	bp_1_9	bp_2_8	bp_3_7	bp_4_6	bp_5_5	bp_6_4	bp_7_3	bp_8_2	bp_9_1
∆_1_	0.1φ	0.2φ	0.3φ	0.4φ	0.5φ	0.6φ	0.7φ	0.8φ	0.9φ
∆_2_	1.9φ	1.8φ	1.7φ	1.6φ	1.5φ	1.4φ	1.3φ	1.2φ	1.1φ

We set DLT = 0.3 and assigned ten different dose-toxicity scenarios (Table 3), setting 5 × 3 dose levels for the trial with a maximum sample size of 60 and an enrollment cohort size of 3. Each scenario was simulated 1000 times. The following metrics were assessed:

1. The percentage of simulated clinical trials in which the correct dose combination was selected as the MTDC (PCS).
2. The percentages of patients at the MTDC (PNMTDC).
3. Average number of DLTs (NDLTS).

**Table 3:** Ten realistic toxicity scenarios with a target toxicity probability of 0.3, where the bold values indicate the maximum tolerated dose combination (MTDC)

Dose Level		Agent 1									
		1	2	3	4	5	1	2	3	4	5
**Agent 2**		**S1**					**S2**				
	1	0.05	0.1	0.15	**0.3**	0.45	0.15	**0.3**	0.45	0.5	0.6
	2	0.1	0.15	**0.3**	0.45	0.55	**0.3**	0.45	0.5	0.6	0.75
	3	0.15	**0.3**	0.45	0.5	0.6	0.45	0.55	0.6	0.7	0.8
		**S3**					**S4**				
	1	0.02	0.07	0.1	0.15	**0.3**	**0.3**	0.45	0.6	0.7	0.8
	2	0.07	0.1	0.15	**0.3**	0.45	0.45	0.55	0.65	0.75	0.85
	3	0.1	0.15	**0.3**	0.45	0.55	0.5	0.6	0.7	0.8	0.9
		**S5**					**S6**				
	1	0.01	0.02	0.08	0.1	0.11	0.05	0.08	0.1	0.13	0.15
	2	0.03	0.05	0.1	0.13	0.15	0.09	0.12	0.15	**0.3**	0.45
	3	0.07	0.09	0.12	0.15	**0.3**	0.15	**0.3**	0.45	0.5	0.6
		**S7**					**S8**				
	1	0.07	0.1	0.12	0.15	**0.3**	0.02	0.1	0.15	0.5	0.6
	2	0.15	**0.3**	0.45	0.52	0.6	0.05	0.12	**0.3**	0.55	0.7
	3	0.3	0.5	0.6	0.65	0.75	0.08	0.15	0.45	0.6	0.8
		**S9**					**S10**				
	1	0.005	0.01	0.02	0.04	0.07	0.05	0.1	0.15	**0.3**	0.45
	2	0.02	0.05	0.08	0.12	0.15	0.45	0.5	0.6	0.65	0.7
	3	0.15	**0.3**	0.45	0.55	0.65	0.7	0.75	0.8	0.85	0.9

*S:* Scenario

**Fig. 3:**
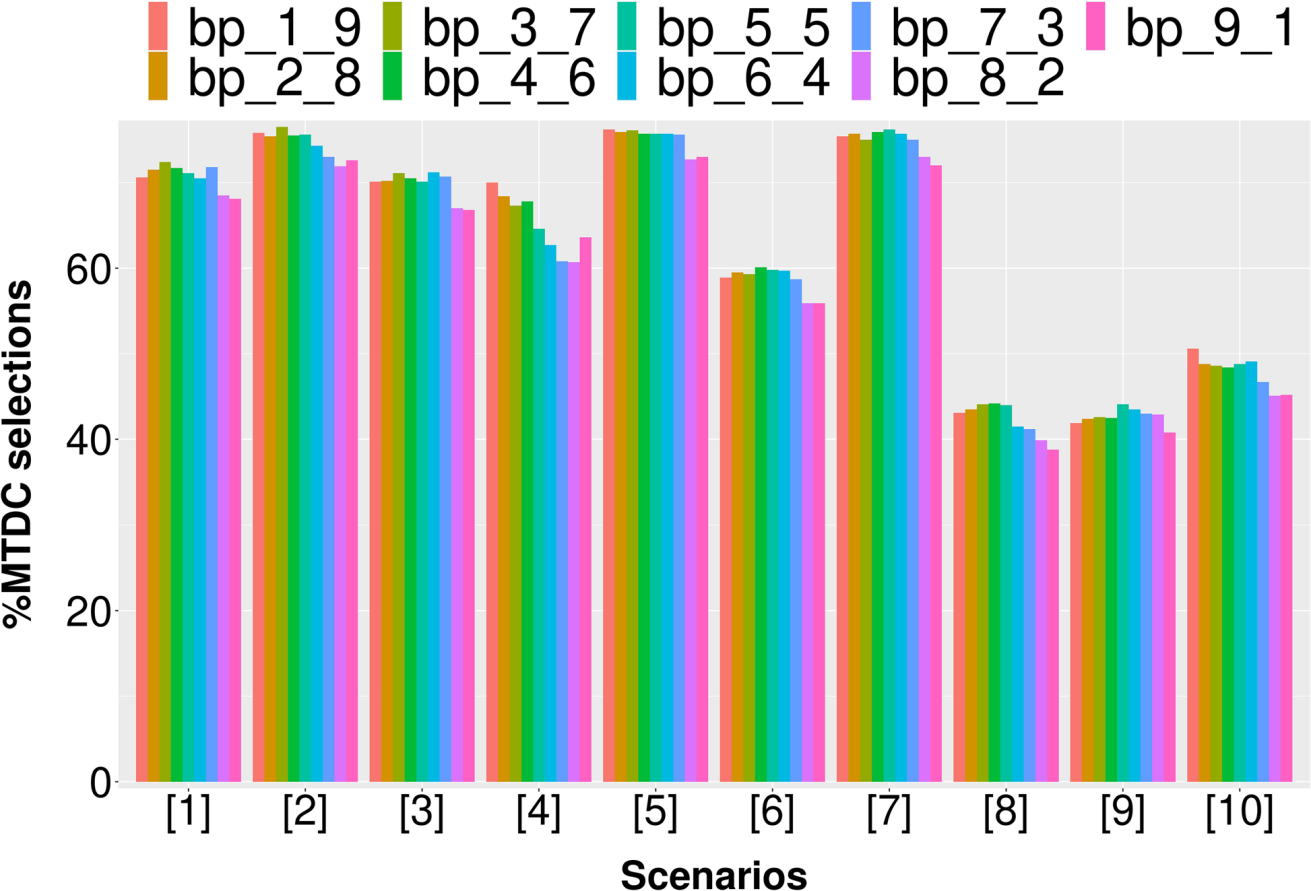
The PCS results of simulation studies with different dose boundary ranges

According to Fig. 3, the first 7 groups of boundaries tend to outperform the last two groups of boundaries in most scenarios when evaluated using the PCS metric. In particular, the first group of boundaries in scenario 4 exhibits a 7.3% higher PCS value compared to the original recommended boundary. Overall, the PCS values of the first 7 groups of boundaries are comparable.

**Fig. 4:**
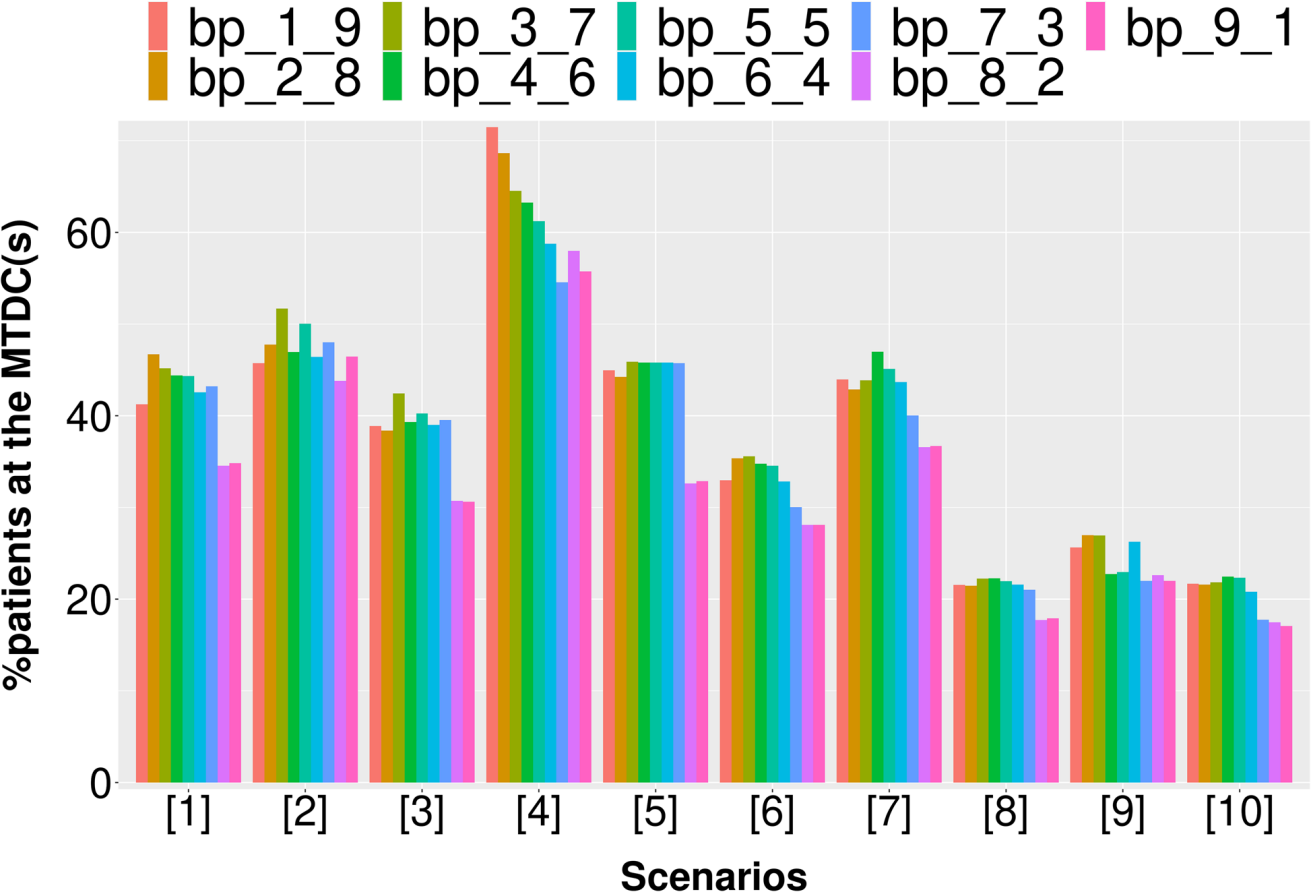
The PNMTDC results of simulation studies with different dose boundary ranges

According to Fig. 4, the first 7 groups of boundaries tend to be better than the last two groups of boundaries in most scenarios when evaluated using the PNMTDC metric. Specifically, the first group of boundaries in scenario 4 exhibits a 13% higher PNMTDC value compared to the original recommended boundaries. Among the first five groups of boundaries, the boundary of group 3 performs particularly well.

**Fig. 5:**
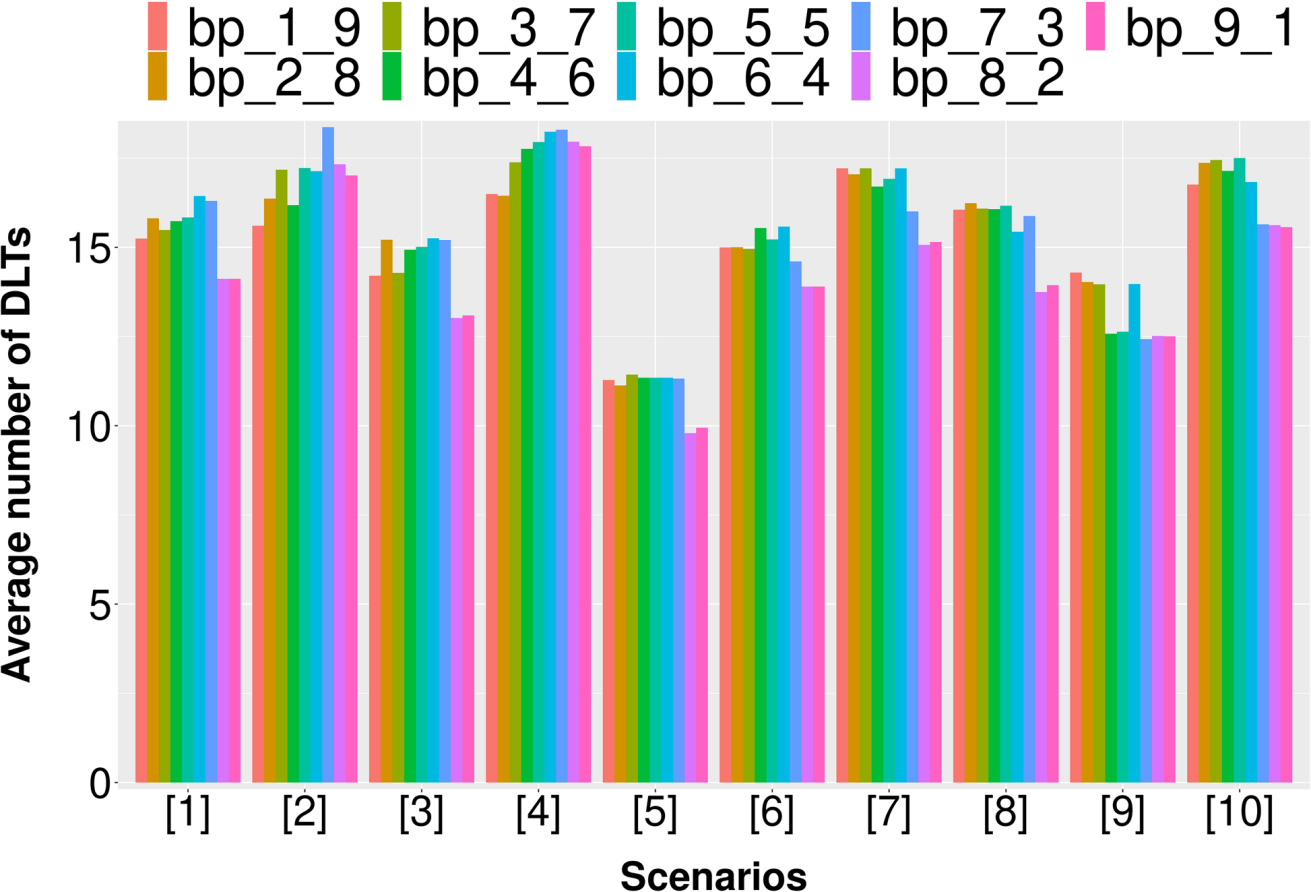
The DLTs results of simulation studies with different dose boundary ranges

Figure 5 indicates that the latter two groups of boundaries often have smaller DLTs, but this is due to a significant decrease in PCS and PNMTD. As a result, these groups are not selected. In general, the first three boundary groups perform better in terms of DLT control and outperform the original recommended boundary group (group 6) in most scenarios. Overall, the design performance under the first three groups of boundaries is better. These boundaries provide more adjustment space for dose escalation and de-escalation, allowing for greater flexibility in dosing and the potential to explore the most suitable method for the current scenario. Additionally, all three indicators PCS, PNMTDC and DLTs are more stable under the boundary of group 3. Therefore, ∆_1_ = 0.3ϕ and ∆_2_ = 1.7ϕ are recommended.

### Simulation studies

After determining the optimal bounding range (∆_1_ = 0.3ϕ, ∆_2_ = 1.7ϕ), we conducted a simulation study of the performance of asBOINcomb and BOINcomb at multiple sample sizes (from 30 to 60 step by 3) using the settings in Section 3.1. The BOINcomb design was implemented using the R package BOIN and with the same bounding range. Each trial continued until the sample was exhausted, unless early termination occurred due to excessive toxicity at the lowest dose combination.

**Fig. 6:**
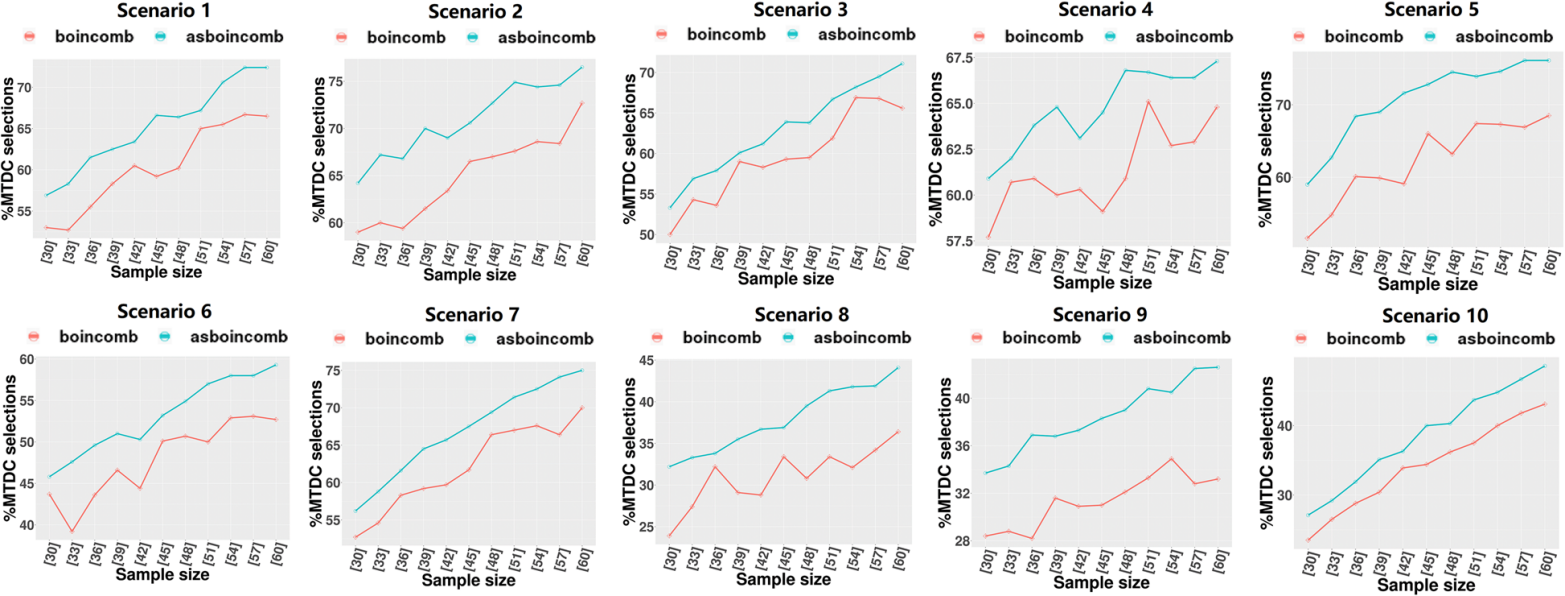
PCS comparison of BOINcomb and asBOINcomb designs for simulation studies with multiple sample sizes

According to Fig. 6, asBOINcomb outperforms BOINcomb when the sample size is 30–60 for all 10 scenarios and converges faster, especially in some extreme scenarios (scenarios 4, 5, 9). For example, in scenario 4, the PCS of asBOINcomb with a sample size of 39 is comparable to that of BOINcomb with a sample size of 51. This demonstrates that asBOINcomb can achieve comparable accuracy to BOINcomb with a smaller sample size, which is beneficial in a clinical context.

**Fig. 7:**
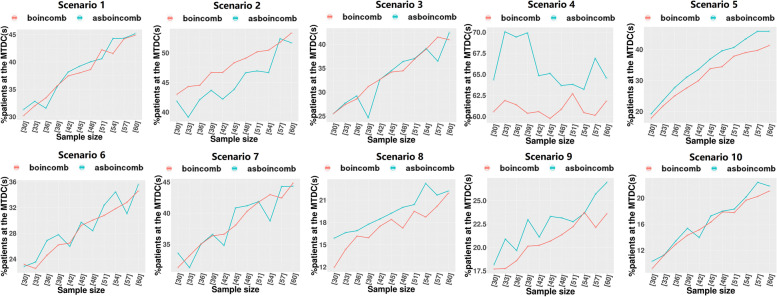
Comparison of PNMTDC of BOINcomb and asBOINcomb designs for simulation studies with multiple sample sizes

According to Fig. 7, asBOINcomb is generally comparable to BOINcomb in terms of PNMTDC when the sample size is between 30 and 60. However, in certain extreme scenarios (scenarios 4, 5, and 9), asBOINcomb performs better. For example, in scenario 4, asBOINcomb exhibits a 8–10% higher PNMTDC than BOINcomb at sample sizes between 33 and 39.

**Fig. 8:**
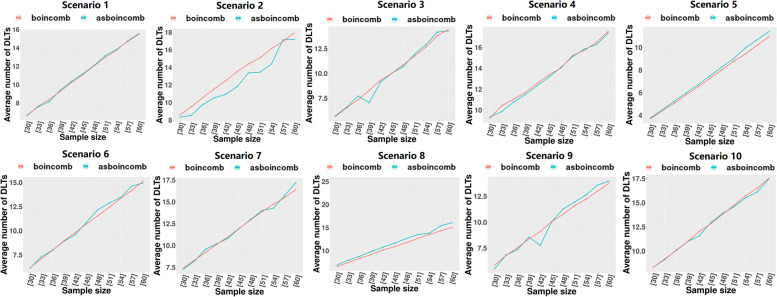
Comparison of DLTs for simulation studies with BOINcomb and asBOINcomb designs at multiple sample sizes

According to Fig. 8, asBOINcomb is generally comparable to BOINcomb in terms of DLTs when the sample size is between 30 and 60. In scenario 2, asBOINcomb exhibits improved DLTs at the cost of a partial decrease in PNMTDC.

Combining the three metrics of PCS, PNMTDC, and DLTs, asBOINcomb has better accuracy than BOINcomb, especially in some extreme scenarios, or equivalently, asBOINcomb can achieve performance comparable to BOINcomb with a smaller sample size.

## Trial example

To further evaluate the proposed strategy, we applied the proposed design to a clinical trial of PF03084014 combined with doxorubicin for the treatment of advanced triple-negative breast cancer (TNBC). PF-03084014 is a reversible, noncompetitive, and selective secretase inhibitor that blocks the NOTCH signaling pathway. In preclinical studies, this combination has demonstrated anticancer efficacy in solid tumor models, such as advanced thyroid cancer and sclerofibrosarcoma, and in T-cell acute lymphoblastic leukemia. In TNBC patient-derived and cell lineage xenograft models, the combination of PF-03084014 with doxorubicin greatly improved the inhibition of tumor growth [27–31].

In this phase I study (A8641016), patients with advanced TNBC were evaluated for safety, tolerability, pharmacokinetics, and antitumor efficacy. The goal of the study was to determine the MTDC of the drug PF03084014 when combined with the chemotherapy drug doxorubicin. The dose exploration component used a modified probability interval technique based on a 2 × 3 matrix design to evaluate toxicity. On day 1 of each 21-day cycle, oral PF-03084014 was coadministered with intravenous doxorubicin twice a day.

To redesign the trial, we fitted logistic regression models to the data observed in this PF03084014 with doxorubicin combination trial. Table 4 illustrates the DLT generated using the estimated probability of toxicity for various combinations of PF03084014 with doxorubicin. This probability reflects a clinician’s estimation of the toxicity of the drug combination.

**Table 4:** Toxicity scenarios for PF03084014 in combination with doxorubicin

	PF03084014		
doxorubicin	1	2	3
**2**	0.22	0.33	0.67
**1**	0.08	0.13	0.36

**Fig. 9:**
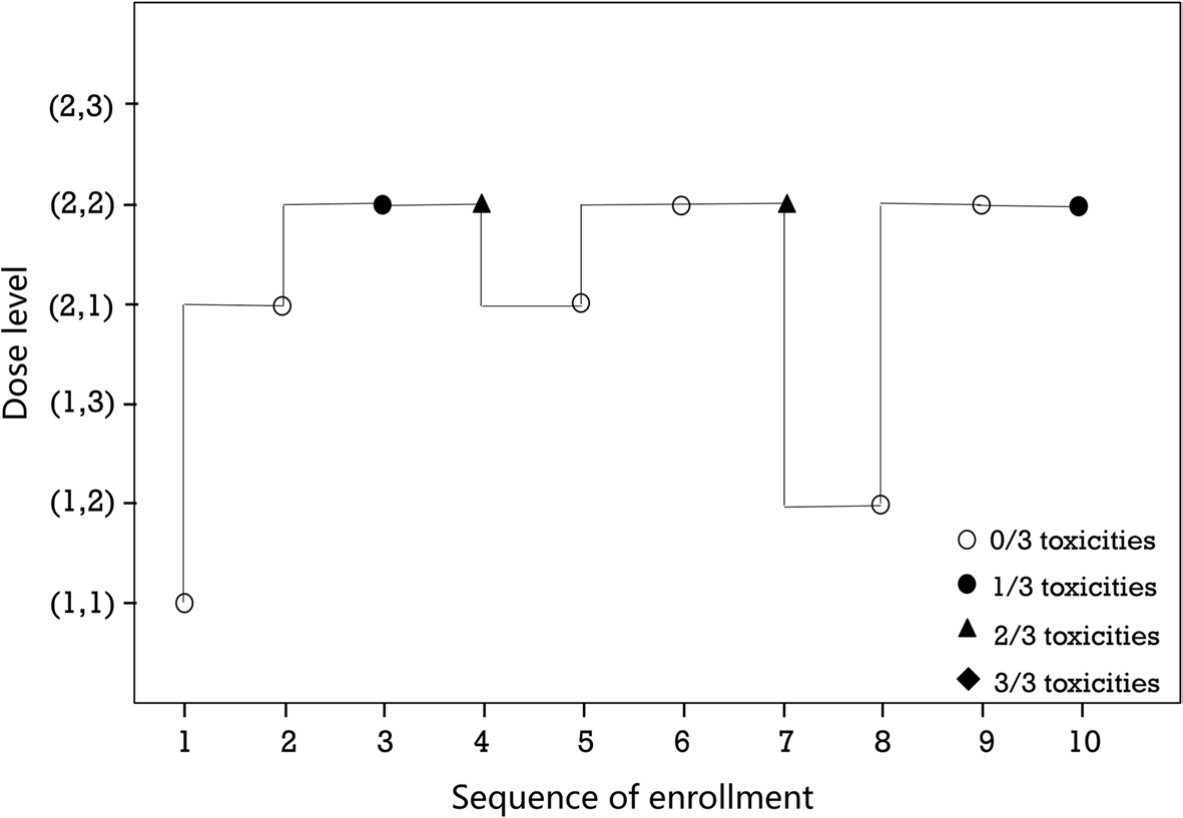
Example of PF03084014 in combination with doxorubicin

The target toxicity probability was set to 0.33, the maximum sample size was 30 patients, the cohort size was 3, and the first group of patients was treated with the lowest dose combination (1, 1). After the parameter set simulation, the optimal parameters *t*_*1*_ = 300 and *t*_*2*_ = 1 were selected as the boundary control conditions for the trial. Figure 9 shows the dose allocation path for the subsequent cohort.

**Fig. 10:**
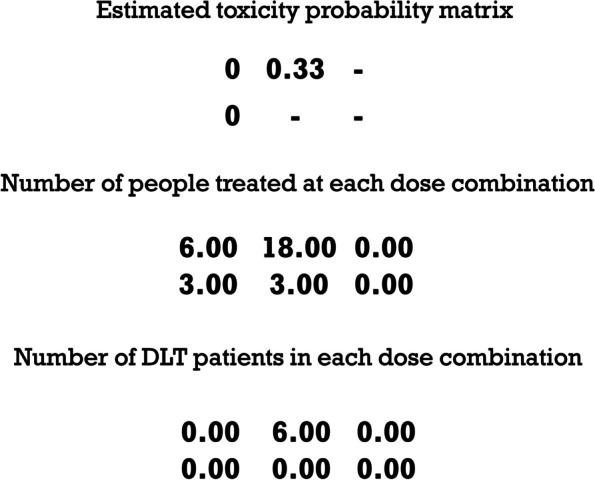
Example results of the PF03084014 and doxorubicin combination trial

As shown in Fig. 9, asBOINcomb can quickly locate the MTDC and treat most patients with the correct dose combination. The estimated toxicity probability matrix at the end of the trial is shown in Fig. 10.

“-” represents a dose combination that was not administered in the trial. The dose combination (2, 2) is selected as the MTDC. As shown in Figs. 9 and 10, the dose levels administered converged to the MTDC starting with 6 sequence of enrollment patients, indicating that the asBOINcomb design can rapidly target the MTDC and treat most patients (60%) with the correct dose combination, while also preventing any patients from being treated at doses above the MTDC. This minimizes the risk of DLT and ensures the safety of the trial.

## Discussion

From statistical and clinical viewpoints, the proposed combination designs are simple and easy to understand. But there are some practical or ethical issues that have not been considered in actual trials. The above design aims to find individual MTDC in the drug combination trial and is not suitable for finding MTDC profiles. This is a topic of our future research.

## Conclusion

We improve the two-dimensional Bayesian optimal interval design by proposing adaptively-shrinking dose escalation/de-escalation boundaries, which converge with increasing sample size. In addition, we introduce the parameter *t*_1_ and *t*_2_ to control the variation trend of the dose escalation/de-escalation boundaries and establish a parameter set to achieve optimal performance. Our simulation results demonstrate that the improved BOINcomb design has better accuracy and stability than the original design, especially in some extreme scenarios. This is largely due to the controllable dynamic boundary which allows for different rates of shrinking for the escalation and de-escalation boundaries. For instance, in highly toxic scenarios, the de-escalation boundary can be set to shrink at a slightly faster rate than the escalation boundary, as stricter or smaller boundaries reduce the risk of exposing subjects to over-toxic doses. The improved accuracy of asBOINcomb means that it can achieve comparable performance to BOINcomb with a smaller sample size, which is highly beneficial in clinical trials. Additionally, the asBOINcomb design allows clinical trial investigators to make decisions based on decision tables, while maintaining the same flexibility and transparency as the BOINcomb design. In conclusion, the proposed design provides a new reference method for Phase I dose finding in subsequent drug combination clinical trials.

## Data Availability

All data generated or analyzed during this study are included in this published article.

## Abbreviations

asBOINcomb: Adaptive shrinking Bayesian optimal interval design for combination drugs
BOINcomb: Bayesian optimal interval design for combination drugs
CRM: Continuous reassessment method
DLT: Dose-limiting toxicity
MTD: Maximum tolerated dose
MTDC: Maximum tolerated dose combination
NDLTS: Average number of DLTs
PCS: Percentage of correct selections
PNMTDC: Percentages of patients at the MTDC
TNBC: Triple-negative breast cancer
